# Molecular characterization and expression of DERL1 in bovine ovarian follicles and corpora lutea

**DOI:** 10.1186/1477-7827-8-94

**Published:** 2010-08-03

**Authors:** Kalidou Ndiaye, Jacques G Lussier, Joy L Pate

**Affiliations:** 1 Department of Animal Sciences, The Ohio State University/Ohio Agricultural Research and Development Center, 1680 Madison Ave, Wooster, OH 44691, USA; 2 Department of Dairy and Animal Science, Pennsylvania State University, University Park, PA 16802, USA; 3 University of Montreal, Faculty of Veterinary Medicine, Saint-Hyacinthe, Quebec J2 S 7C6, Canada; 4 Department of Anatomy and Physiology, Kansas State University, 1600 Denison Ave, Manhattan, KS 66506, USA; 5 Department of Dairy and Animal Science, Pennsylvania State University, University Park, PA 16802, USA

## Abstract

The endoplasmic reticulum (ER) is a major site of protein synthesis and facilitates the folding and assembly of newly synthesized proteins. Misfolded proteins are retrotranslocated across the ER membrane and destroyed at the proteasome. DERL1 is an important protein involved in the retrotranslocation and degradation of a subset of misfolded proteins from the ER. We characterized a 2617 bp cDNA from bovine granulosa cells that corresponded to bovine DERL1. Two transcripts of 3 and 2.6 kb were detected by Northern blot analysis, and showed variations in expression among tissues. During follicular development, DERL1 expression was greater in day 5 dominant follicles compared to small follicles, ovulatory follicles, or corpus luteum (CL). Within the CL, DERL1 mRNA expression was intermediate in midcycle, and lowest in late cycle as compared to early in the estrous cycle. Western blot analyses demonstrated the presence of DERL1 in the bovine CL at days 5, 11, and 18 of the estrous cycle. Co-immunoprecipitation using luteal tissues showed that DERL1 interacts with class I MHC but not with VIMP or p97 ATPase. The interaction between DERL1 and MHC I suggests that, in the CL, DERL1 may regulate the integrity of MHC I molecules that are transported to the ER membrane. Furthermore, the greater expression of DERL1 mRNA is associated with the active follicular development and early luteal stages, suggesting a role of DERL1 in tissue remodeling events and maintenance of function in reproductive tissues.

## Background

The endoplasmic reticulum (ER) is the first compartment of the secretory pathway and is a processing station for secreted and transmembrane proteins. The primary function of the ER is to assist newly synthesized proteins to refold into native conformation. To achieve correct folding and maturation, secreted proteins must translocate into the ER to undergo several post-translational modifications, including glycosylation and disulfide bonding [1]. Proteins that fail to fold properly in the ER are recognized by the quality control machinery and transferred from the ER into the cytosol to the ubiquitin-proteasome system in a process called retrotranslocation or dislocation [2]. The ER-associated degradation (ERAD) machinery serves as one part of the adaptive cellular program to destroy the potentially toxic accumulation of misfolded proteins. A dysfunction in the ERAD pathway causes human diseases [3] while many viral proteins can hijack this pathway to evade detection by the immune system [4].

This latter phenomenon is used by the human cytomegalovirus (HCMV) to eliminate class I major histocompatibility complex (MHC I) molecules to distract cytotoxic T cells from destroying infected cells [5]. The HCMV encodes the glycoproteins US2 and US11 that induce retrotanslocation of newly synthesized class I heavy chains [6]. The HCMV US2 and US11 proteins utilize the normal ER quality control process to eliminate class I heavy chains in a similar manner as misfolded or damaged ER proteins. The ERAD of class I MHC heavy chain by HCMV-encoded US11 involves DERL1, a mammalian ER membrane protein, homolog of Der1p in *Saccharomyces cerevisiae *that is essential for the relocation of misfolded proteins from the ER lumen into the cytosol [7,8]. This action of DERL1 is performed in concert with the ATPase p97/Valosin-containing protein (VCP) that forms a complex with DERL1 [7,8] after being recruited by the VCP-interacting membrane protein (VIMP), which can interact with DERL1 [8].

The expressions of class I and class II MHC molecules have been demonstrated in the corpus luteum (CL) where they may function to regulate T lymphocyte activation, thus controlling the production of cytokines [9-13]. The CL is a very heterogeneous population of cells that becomes rapidly organized into a functional unit. These diverse cells communicate both directly and through paracrine mediators to facilitate the steroidogenic function and the transient nature of the CL. The CL also contains nonsteroidogenic cells, including endothelial cells, fibroblasts, and immune cells that have a role in luteal function by communicating with steroidogenic cells through the paracrine signaling molecules they produce and through direct cell contacts [14,15]. The MHC molecule-dependent interaction between luteal cells and T cells is one form of direct cell-cell signaling that may serve to activate resident immune cells [16]. An association between MHC proteins and the ERAD proteins in reproductive tissues has not been previously investigated. Thus, studying the link between class I MHC and DERL1 in the CL may contribute to understanding the control of luteal function.

There has been significant interest in the role of DERL1 in the ER quality control machinery. However, nothing is known about the expression of DERL1, its regulation and physiological relevance in reproductive tissues, such as the ovary. In this study, the expression of DERL1 in bovine granulosa and luteal cells, and its regulation at different stages of follicular growth and corpora lutea development were analyzed. Using normal luteal tissue extracts, putative interactions of DERL1 with MHC I, p97 ATPase, and VIMP were also investigated.

## Methods

### Animal model for follicle collection

The regulation of *DERL1 *mRNA expression during follicular development and ovulation was studied using *in vivo *models as previously characterized [17]. Briefly, estrous cycles of normally cyclic crossbred heifers were synchronized with one injection of prostaglandin F_2 _alpha (PGF_2α_; 25 mg, im; Lutalyse, Upjohn, Kalamazoo, MI) given in the presence of a CL. Ovarian follicular development was monitored by daily transrectal ultrasonography. Following estrous synchronization, heifers were randomly assigned to the dominant follicle group (DF, n = 4), or the ovulatory, hCG-induced follicle group (OF, n = 4). Immediately following ovariectomy, granulosa cells (GC) were collected separately from individual DF or OF as described previously [17], and stored at -70°C. Additionally, GC were collected from 2-4 mm small follicles (SF) obtained from slaughterhouse ovaries, and a total of three pools of 10-20 SF was prepared. Corpora lutea at day 5 of the estrous cycle were obtained by ovariectomy and were dissected from the ovarian stroma, frozen in liquid nitrogen, and stored at -70°C until RNA was extracted. The Animal Ethics Committee of the Faculty of Veterinary Medicine of the University of Montreal approved all animal procedures.

### Corpora lutea removal

Corpora lutea were collected transvaginally from cyclic cows at early (day 5) mid (day 11) and late (day 18) luteal phase of the estrous cycle (day of estrus = day 0) and at 1, 4, 8, and 12 hours after a 25-mg injection of PGF_2α _(Lutalyse; Upjohn, Kalamazoo, MI) in the midluteal phase to induce luteal regression. Total RNA was extracted from luteal tissues to quantify *DERL1 *mRNA concentrations by real time-quantitative PCR. Corpora lutea collection was performed at the Ohio State University and handling of animals and surgical procedures were conducted according to protocols approved by the Institutional Laboratory Animal Care and Use Committee of the Ohio State University.

### Cloning of bovine DERL1

The *DERL1 *cDNA was cloned from a bovine cDNA library prepared with polyA^+ ^mRNA isolated from granulosa cells of dominant follicles at day 5 of the estrous cycle. The cDNA library was constructed in lambda Zap Express vector (Stratagene, La Jolla, CA) by unidirectional cloning of cDNAs as described [18]. Following *in vivo *excision of pBluescript phagemids containing the cloned cDNA insert with the Ex-Assist/XLOLR system (Stratagene), single bacterial colonies were picked and their phagemid content were purified by mini-prep (Qiagen, Mississauga, ON). The cDNA inserts were analyzed by sequencing, and sequences were compared by BLAST in GenBank database. Alignment of deduced amino acid sequences of bovine DERL1 protein with putative homologs from other organisms was performed using the ClustalW2 analysis software (EMBL-EBI, Hinxton, Cambridge, UK). *DERL1 *cDNA was entirely characterized by sequencing on an ABI Prism 310 (Applied BioSystem).

### RNA isolation and northern blot analysis

Various bovine tissues were obtained from a local slaughterhouse and RNA was extracted in lysis buffer (for 500 ml buffer: 4 M guanidium isothiocyanate, 2.5 ml Na-N-laurylsarcosine, 25 mM Na-citrate, pH 7), and sedimented, following centrifugation on a cesium chloride cushion as described [19]. The concentration of total RNA was quantified by measurement of optical density at 260 nm, and RNA integrity was evaluated by visualizing the 28 S and 18 S ribosomal RNA bands following electrophoretic separation on 0.66 M formaldehyde denaturing 1% agarose gel with ethidium bromide. Total RNA (20 μg/tissue) were size-fractionated on a 0.66 M formaldehyde, 1% agarose gel, transfered by capillarity to a nylon membrane (Hybond-N; Amersham Pharmacia Biotech), and UV-treated (150 mJ) as described [20]. The bovine *DERL1 *cDNA was used to generate a radioactive probe incorporating [α^32^P]-dCTP (NEN Life Sciences, Boston, MA) that was used to hybridize Northern blots as described [19].

### Alternative splicing analysis of bovine DERL1

Analysis for the presence of putative alternative splicing variants for bovine *DERL1 *mRNA was performed by RT-PCR using total RNA from bovine CL. The first step consisted of a reverse transcription reaction performed with 1 μg of total RNA using the M-MLV Reverse Transcriptase (Promega) following the manufacturer's protocol. The second step consisted of a PCR amplification with specific primers (foward: 5'-ATGTCGGACATCGGGGACTGG-3'; reverse: 5'-CTGGTCCCCGAGCCGAAAGC-3') located respectively at the start codon and stop codon of the *DERL1 *open reading frame (ORF) amplifying the entire ORF of 754 bp in a 35 cycle PCR reaction. The PCR products were analyzed for size and specificity using the Agilent DNA 1000 kit on the Agilent 2100 BioAnalyzer (Agilent Technologies).

### Semi-quantitative RT-PCR

Expression of *DERL1 *mRNA during follicular development was analyzed by semi-quantitative RT-PCR. Total RNA was extracted from bovine granulosa cells (GC) collected from 2-4 mm small follicles (SF), dominant follicles at day 5 of the estrous cycle (DF), ovulatory follicles 24 hours after an injection of hCG (OF), and from day 5 corpora lutea (CL). *DERL1 *PCR primers were as follows: (forward: 5'-TATCGCTTCCAGATTTGGAGG-3'; reverse: 5'-GAATCCAAGGATAACCCAAGG-3'). The number of cycles was limited and optimized for analysis of *DERL1 *mRNA expression. PCR reaction products were separated on a 2% TAE-agarose gel with ethidium bromide, visualized by UV light, digitized and analyzed by densitometry using ImageQuant software (Amersham Pharmacia Biotechniques). *GAPDH *was used as a control gene, and specific signals of *DERL1 *were normalized with corresponding *GAPDH *signals.

### qPCR analysis

Total RNA was extracted from luteal tissues using Trizol reagent as described [15], quantified using the Nanodrop spectrophotometer (Thermo Scientific), and treated with RNase-free DNase I (Roche Molecular Biochemicals) to eliminate genomic DNA contamination. qPCR was used to detect and quantify *DERL1 *mRNA. Oligonucleotide primers specific for *DERL1 *transcript, described in the semi-quantitative RT-PCR section, were used to amplify *DERL1 *cDNA. Ribosomal protein L19 (*RPL19*) cDNA fragment was amplified as a constitutively expressed gene with the following primers (forward: 5'-ATCGATCGCCACATGTATCA-3'; reverse: 5'-GCGTGCTTCCTTGGTCTTAG-3'). Total RNA (1 μg) was reverse transcribed using the iScript cDNA Synthesis Kit (Bio-Rad Laboratories) according to the protocol of the manufacturer. Following the reverse transcription reaction, quantitative PCR was performed on the MJ Research Opticon 2 (Bio-Rad Laboratories) as described [15] using the iQSYBR Green Supermix (Bio-Rad Laboratories). Homologous standard curve prepared from purified *DERL1 *cDNA PCR product was used to calculate the relative steady-state concentrations of *DERL1 *mRNA in triplicate wells for each sample. The PCR amplification products were electrophoretically separated on 1.5% agarose gels and visualized with ethidium bromide. For initial validation, the specific band corresponding to the size of the expected *DERL1 *cDNA fragment was cut and purified using the QIAquick Gel Extraction Kit (Qiagen Sciences) for sequence confirmation. A control sample that was not reverse transcribed was used to confirm that the product obtained was not amplified from genomic DNA.

### Protein extraction and immunoblotting

Proteins were extracted from luteal tissues at days 5, 11, and 18 of the estrous cycle using the CelLytic MT Cell Lysis Reagent (Sigma-Aldrich Biotechnology) in the presence of the protease inhibitor cocktail (Sigma-Aldrich Biotechnology) following the manufacturer's protocol. Proteins were quantified according to the method of Bradford [21] (Bio-Rad Protein Assay, Bio-Rad Laboratories). Protein samples (75 μg) were subjected to electrophoresis on a 12% SDS-polyacrylamide gel, and the separated proteins were blotted onto polyvinylidene difluoride membranes (PVDF; Hybond-P, Amersham Pharmacia Biotech). Western blot analyses were performed exactly as described [15] using a polyclonal rabbit anti-DERL1 antibody (NB100-447SS; Novus Biologicals), raised against a synthetic peptide corresponding to amino acid residues 150-250 of human DERL1 at a final concentration of 0.5 μg/ml. Membranes were then washed as described [15] and incubated with the horseradish peroxidase-labeled anti-rabbit secondary antibody (Amersham Biosciences) at a dilution of 1:20 000. The antigen-antibody complex was visualized using the enhanced chemiluminescence system (ECL Western Blotting Analysis System; Amersham Biosciences) following the manufacturer's protocol. Membranes were exposed to Kodak Biomax light films (Kodak), and the films were developed in the SRX-101A Konica film processor (Konica Corporation, Japan). Protein bands were analyzed by densitometry using the Kodak Image Station 4000R software (Eastman Kodak Co.). Beta actin was used as an internal control to verify the integrity of proteins in the samples and specific signals of DERL1 were normalized with corresponding beta actin signals.

### Co-immunoprecipitation

Protein samples were obtained from luteal tissues as described above. Immunoprecipitation of DERL1 was performed following the protocol as described [22]. Protein lysates (100 μg) were first incubated with 100 μl of *S. aureus *Cowan I (Pansorbin, Calbiochem) saturated with normal rabbit serum and 50 μl of Sepharose CL-4B (Amersham Pharmacia Biotech). After an overnight incubation at 4°C with continuous gentle rocking followed by centrifugation (10,600 g, 15 min, 4°C), the supernatant was collected and incubated with 15 μl of specific anti-DERL1 antibodies with rocking for 2 hours at 4°C. The antigen-antibody complex was precipitated by the addition of 80 μl of protein A Sepharose CL-4B (Amersham Pharmacia Biotech) for 1 hour at 4°C with rocking followed by centrifugation (10,600 g, 1 min, 4°C) to pellet the complex. Supernatants were collected at this point and stored at -70°C for further analysis. The pelleted precipitates containing DERL1 and potential bound proteins were washed 3 times in lysis buffer containing 1% Nonidet-P40. Both precipitate and corresponding supernatant samples were heated at 90°C for 5 min in the presence of Laemmli loading buffer and separated on SDS-polyacrylamide gel alongside molecular weight markers (Bio-Rad Laboratories). Proteins were transferred onto PVDF membranes and blotted separately with specific antibodies against class I MHC (H17A; VMRD), VIMP (V6639; Sigma), and p97 (PRO65278; Research Diagnostics Inc.). Additional anti-DERL1 antibodies were obtained from Sigma (D4443) in order to confirm the presence of DERL1 in the precipitates.

### Statistical analysis

For the data from semi-quantitative PCR experiments, gene-specific signals were normalized with corresponding *GAPDH *signals for each sample. Homogeneity of variance among follicular groups and CL was verified by O'Brien and Brown-Forsythe tests. Corrected values of gene-specific mRNA were compared by one-way ANOVA in GC obtained from bovine follicles collected at different developmental stages or CL. When ANOVA showed significant differences, Tukey-Kramer multiple comparisons was used to determine differences at p < 0.05. The same statistical procedure was used for the western blot data with specific DERL1 signals normalized with corresponding beta actin signals for each sampling day. All other statistical analyses were performed using the covariate analysis within the mixed model of SAS (SAS Inst. Inc., Cary, NC) with *RPL19 *as the covariate. Data were presented as least-square means ± SEM and differences were considered significant at p < 0.05.

## Results

### Cloning of bovine DERL1

The cDNA of *DERL1 *was cloned from bovine granulosa cells (GC) of day 5 dominant follicles and characterized by sequencing. The cDNA of *DERL1 *is 2617 bp in length and encodes a hydrophobic protein of about 28.8 kDa containing 251 amino acid residues (GenBank: AF279909). Amino acid analysis indicated that DERL1 presents four putative transmembrane domains located between amino acid residues F^19^-S^35^, L^99^-A^115^, L^120^-L^136^, and L^155^-I^171^, a feature of membrane protein. Sequence comparison showed orthologous proteins from human (GenBank accession number: NP_077271), pig (XP_001928865), mouse (NP_077169), chicken (NP_001006350), and frog (NP_001085401) that were, respectively, 99, 99, 97, 90, and 86% identical to bovine DERL1 (AF279909).

### DERL1 mRNA expression and regulation during follicular development

**Figure 1:**
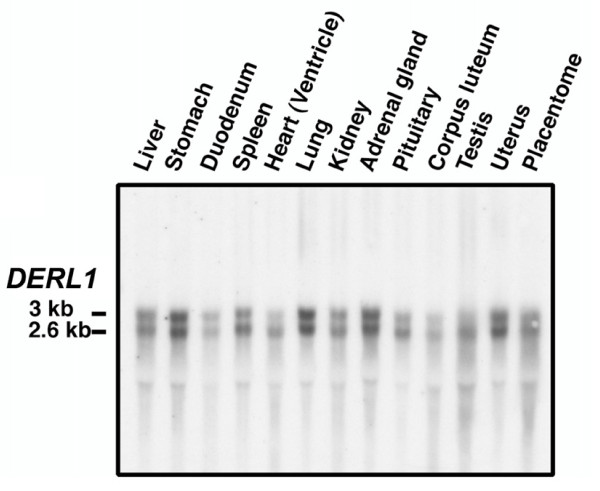
**Analysis of *DERL1 *mRNA expression in bovine tissues by Northern blot**. Two transcripts of 3 and 2.6 kb corresponding to bovine *DERL1 *were observed and showed a variable pattern of expression among tissues.

**Figure 2:**
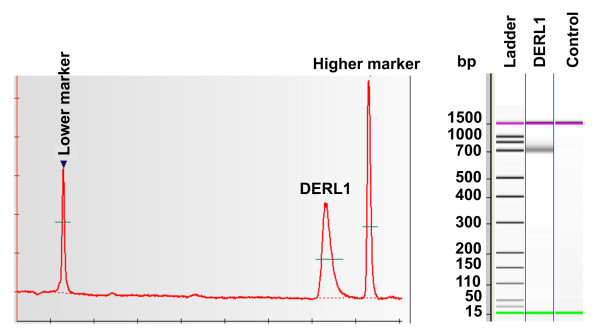
**Analysis of alternative splicing of *DERL1 *mRNA**. The specific *DERL1 *PCR product corresponding to the entire open reading frame is represented by a single *DERL1 *PCR product; the sample without RT enzyme control shows that the specific *DERL1 *product amplified is not from genomic DNA.

The expression of *DERL1 *mRNA was first analyzed via Northern blot using several bovine tissues. Two transcripts of *DERL1 *were detected at 3 kb and 2.6 kb (Fig. 1). *DERL1 *mRNA expression was observed in all tissues analyzed, with variable intensities among tissues. The *DERL1 *mRNA expression was greatest in adrenal gland, lung and uterus, and was moderate in other tissues including the CL. To verify possible alternative splicing of *DERL1 *mRNA, PCR experiments amplifying the entire open reading frame of *DERL1 *were performed and demonstrated a single product (Fig. 2). No amplification was observed in the control (without reverse transcriptase enzyme) (Fig. 2) showing that the product amplified is not from genomic DNA. This result suggests that one protein is encoded from the mRNA and that the two transcripts observed by Northern blot might differ at their 3'-untranslated region. This difference could potentially mean a different control of the stability of *DERL1 *mRNA.

**Figure 3:**
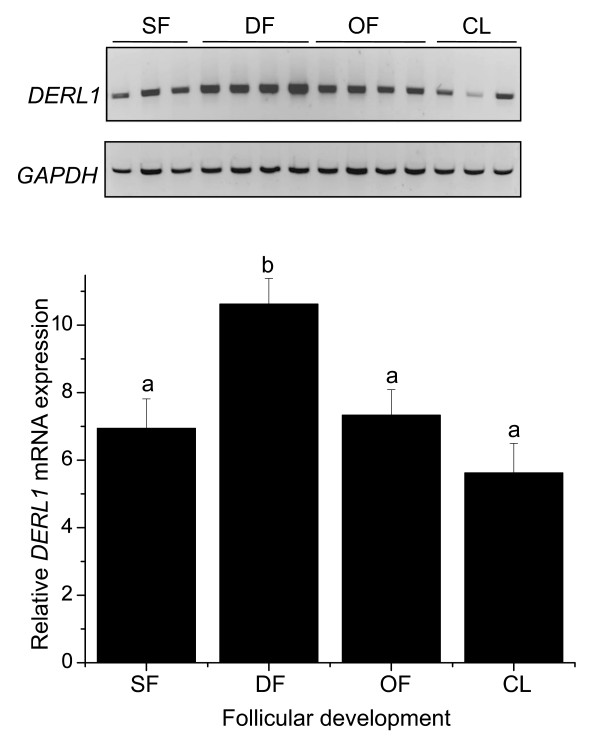
**Analysis of *DERL1 *mRNA expression during follicular development by semi-quantitative RT-PCR**. The control gene was *GAPDH *and showed no significant differences in mRNA expression among the different groups. The *DERL1 *PCR signals were normalized with their corresponding *GAPDH *signals, and the results are presented as a relative change in ratio among groups. Different letters denote samples that differed significantly (*p *< 0.05) when Tukey-Kramer multiple comparison tests were performed to compare group means. Data are presented as means ± SEM. DF = dominant follicles (n = 4); OF = ovulatory follicles (n = 4); SF = 2-4 mm small follicles (n = 3); CL = corpus luteum collected on day 5 of the estrous cycle (n = 3).

The regulation of *DERL1 *mRNA during follicular development was analyzed using semi-quantitative RT-PCR. Greater expression of *DERL1 *was observed in granulosa cells obtained from day 5 dominant follicles (D5) compared to small follicles (SF), ovulatory follicles (OF), and day 5 corpus luteum (CL) (p < 0.05; Fig. 3). There was no difference in *DERL1 *mRNA among SF, OF, and CL (Fig. 3). *GAPDH *used as control gene was not different among groups (p < 0.43).

### DERL1 mRNA expression in bovine corpus luteum

**Figure 4:**
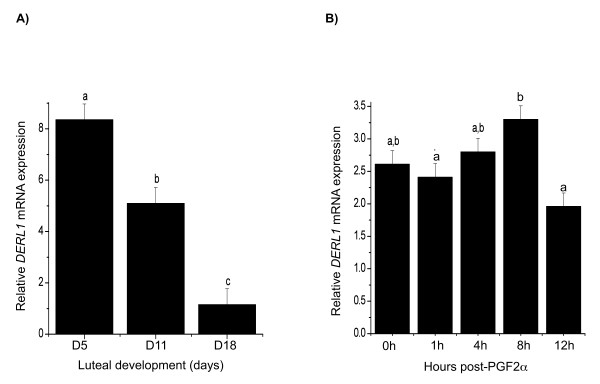
**A) RT-PCR detection of *DERL1 *mRNA expression in bovine corpus luteum at different stages of luteal development and B) at different hours post-PGF**_**2α **_**(0, 1, 4, 8, and 12 hours)**. The control CL (0 h) was obtained on day 11 of the estrous cycle. Different letters denote significant differences (p < 0.05). Data are presented as means ± SEM; n = 4 animals at each day or time point.

RT-qPCR experiments using total RNA from luteal tissues showed that steady state concentration of *DERL1 *mRNA was intermediate in midcycle (day 11, p = 0.064), and lowest in late cycle (day 18, p < 0.05) as compared to early in the estrous cycle (day 5) (Fig. 4A). In addition, *DERL1 *in day 11 CL was greater than in day 18 CL (p < 0.05). Injection of PGF_2α _during the midcycle induced a transient increase of *DERL1 *mRNA in the CL after 8 hours (p = 0.065) followed by a slight decrease at 12 hours post PGF_2α _(p = 0.081), compared to day 11 CL (Fig. 4B). Additionally, *DERL1 *mRNA was greater at 8 hours post PGF_2α _than at 1 hour and at 12 hours (p < 0.05; Fig. 4B).

### DERL1 protein expression

**Figure 5:**
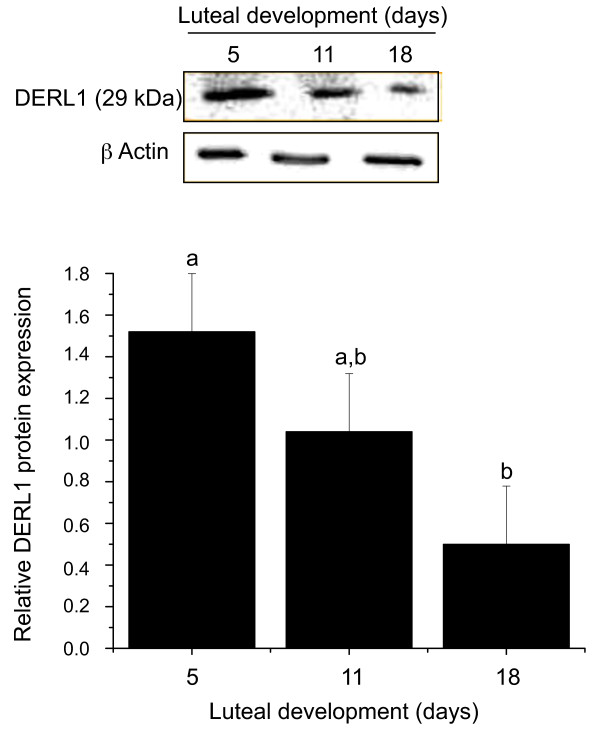
**Representative Western blot analyses using antibodies against DERL1 for protein expression in bovine corpus luteum**. DERL1 protein was observed in bovine CL at day 5, day 11, and day 18 of the estrous cycle, with corresponding β actin blots. The experiment was repeated using CL protein lysates from four animals with similar results. The histogram in the lower panel represents the quantitative expression of DERL1 during luteal development.

In order to analyze the expression of DERL1 protein in the CL, western blot analyses were performed and demonstrated the presence of DERL1 in luteal tissues from days 5, 11, and 18 of the estrous cycle (Fig. 5). Similar to the mRNA, DERL1 protein expression was greater in day 5 CL as compared to day 18 CL. Beta actin used as the control protein did not show any variation throughout the estrous cycle.

**Figure 6:**
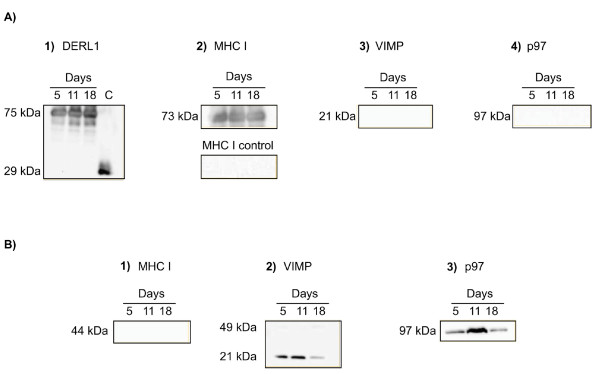
**Co-immunoprecipitation of DERL1 and bound proteins**. A) Western blotting using the pelleted precipitates from immunoprecipitation. Blots were incubated, separately, with anti-DERL1 (1; C = DERL1 control), anti-MHC I (2), anti-VIMP (3), and anti-p97 (4) antibodies. B) Western blotting using the supernatant collected following co-immunoprecipitation. Blots were incubated with anti- MHC I (1), anti-VIMP (2), and anti-p97 (3) antibodies.

To determine if DERL1 interacts with class I MHC, VIMP, or p97 ATPase, immunoprecipitation was performed using protein lysate from luteal tissues with anti-DERL1 antibodies from Novus Biologicals (NB100-447SS) followed by western blotting and incubation with various antibodies. Incubation with anti-DERL1 antibodies from Sigma (D4443) showed the presence of DERL1 in the control sample from day 5 CL at the predicted molecular weight of about 28 kDa, and a second band was observed at around 75 kDa in the precipated samples from days 5, 11, and 18 of the estrous cycle (Fig. 6A1) representing DERL1 and an interacting protein. Class I MHC antibodies used with the same precipitates on a separate blot recognized a band of about 73 kDa (Fig. 6A2, top panel). To verify that class I MHC did not bind to protein A used for precipitation, class I MHC antibodies were incubated with blots from a mixture containing only protein A with an aliquot of protein lysate, in the absence of DERL1 antibodies. This experiment showed no binding of protein A to class I MHC (Fig. 6A2, bottom panel), confirming that the presence of class I MHC in the precipitate is due to its interaction with DERL1. Furthermore, the size of the band observed in the precipitates using both anti-DERL1 and anti-MHC I indicates that it is a result of the interaction between DERL1 and MHC I. Finally, MHC I protein was absent in the supernatant collected after precipitation with anti-DERL1 antibodies and protein A (Fig. 6B1). In contrast, there was no interaction of DERL1 with VIMP or p97 in the CL at any day of the estrous cycle (Fig. 6A3, A4). However, both VIMP and p97 were detected in the supernatant using, respectively, specific anti-VIMP and anti-p97 antibodies (Fig. 6B2, B3). Two bands of around 21 kDa and 49 kDa were observed for VIMP in the supernatant (Fig. 6B2).

## Discussion

These results provide support for a physiologically relevant role for DERL1 in the ovary. First, the greatest expression of *DERL1 *is associated with active ovarian follicular growth, suggesting a role of DERL1 in developing follicles. The preovulatory LH surge induces several changes in different compartments of the ovarian follicle as it triggers ovulation, maturation of the cumulus oocyte complex, and luteinization [23-26]. In ovulatory follicles, *DERL1 *decreased compared to dominant follicles in our *in vivo *study. Following the comparison of its mRNA profile in different bovine tissues by Northern blot analysis, we hypothesized that *DERL1 *mRNA in granulosa cells would vary in relation to follicular development. Indeed, we have shown that *DERL1 *mRNA in granulosa cells is regulated depending on the developmental stage of the follicle. Increased expression of *DERL1 *in dominant follicles and early CL might contribute to protect granulosa cells against apoptosis and/or developmental stresses during follicular or luteal growth. Similarly, recent data in breast cancer cells showed that DERL1 expression is increased by ER stress while DERL1 knockdown resulted in decreased development of cancer cells [27]. The latter study demonstrated that DERL1 expression might be induced by stress inducers in breast cancer cell lines and suggested that DERL1 could protect cancer cells against ER stress-induced apoptosis [27]. These observations support a crucial role of DERL1 for survival of rapidly growing cells within normal tissues such as granulosa cells or within tumorigenic tissues.

Additional evidence for the physiological significance of DERL1 in the ovary is the differential expression of DERL1 mRNA and protein during the luteal phase, and the physical interaction of DERL1 protein with class I MHC in freshly collected luteal tissues. No report is available regarding DERL1 expression and regulation in the CL. We demonstrated a link between DERL1 expression and the early stages of the functional CL. Following ovulation, the luteinization process is realized under intense tissue remodeling and organization. This process involves repression and/or expression of several factors. The greatest expression of DERL1 early in this process is in accordance with its putative role in the quality control machinery of newly synthesized proteins. Toward the end of the estrous cycle, DERL1 mRNA and protein decreased significantly, suggesting that DERL1 might be required for the maintenance of a functional CL. Analysis of progesterone (P_4_) concentrations showed that the CL used at day 18 were not regressing further supporting that the decrease in DERL1 expression was a physiological event.

The exact role of DERL1 in the ovary is unknown. However, as previously hypothesized for other systems [7,8], DERL1 proteins may form a channel by oligomerization or association with other factors, and are required for protein degradation associated with the ER. Also, it has been suggested that p97 ATPase participates in a complex with DERL1 and that, in this complex, p97 ATPase provides the energy for dislocation [28]. In the CL, we did not find any interaction between DERL1 and p97. This may be because this interaction is triggered by a specific event in the cell such as ER stress due to accumulation of misfolded or unassembled proteins. An early step during the assembly of a retrotranslocation complex involves the targeting of a retrotranslocation polypeptide to DERL1 that is not associated with the p97 ATPase [29]. At a later stage, the p97 ATPase joins DERL1 to form a larger retrotranslocation complex. Similarly, in the present study, no interaction was found between DERL1 and VIMP. The membrane protein VIMP was shown to interact with DERL1 and recruits the p97 ATPase and its cofactor, the UFD1/NPL4 complex, to the ER for retrotranslocation of misfolded proteins into the cytosol [8]. The absence of interaction between DERL1 and VIMP would then explain why there is no recruitment of the p97 ATPase in luteal tissues.

Interestingly, DERL1 was found to interact with class I MHC molecules in luteal tissues used in the present study. A physiological role for MHC molecules in luteal function is supported by the fact that both class I and class II molecules are expressed by luteal cells *in vivo *[10-12,30]. MHC molecules mediate the interaction between luteal cells and T lymphocytes as a form of cell-cell signaling to activate resident immune cells [16]. The expression of MHC molecules and the recognition of antigenic peptides by MHC serve as a means to regulate T lymphocyte activation, thus controlling cytokine production. DERL1 interacts with US11, a virally-encoded ER protein that specifically targets MHC class I heavy chains for export from the ER [8,31-33]. In the present study, normal luteal tissues, analyzed at different stages of development, showed a constant and direct interaction between DERL1 and class I MHC. As a DERL1-associated protein, class I MHC may represent a factor that targets dislocation of substrates to the multiprotein complexes. Alternatively, binding of DERL1 with class I MHC may prevent presentation of processed peptides to T lymphocytes, because DERL1 will control the integrity of class I molecules, preventing activation of T lymphocytes. Stimulation of T lymphocyte activation is dependent on the specific interaction of a T cell receptor for antigen (TCR) on the T cell with MHC molecules located on the surface of the target cell [34]. The outcome of binding of the TCR to the MHC molecule is determined by the peptides bound to the MHC molecules. Antigenic peptides that are presented to T cells via MHC molecules are derived from proteins that are proteolytically digested into short peptides prior to binding to MHC molecules. Since antigen processing can determine the types of peptides bound to MHC molecules, this process can impact whether cells within a tissue are able to activate T lymphocytes, thereby stimulating an immune response, while binding of DERL1 to class I MHC would affect this process.

## Conclusions

The ability of cells to dispose of misfolded proteins is critical for cell survival. The expression of DERL1 in the developing follicles and in the functional CL provides strong evidence that DERL1 might be involved in tissue remodeling events and maintenance of function. Although the exact function of DERL1 in the ovary is not fully understood, its greater expression seems to be specifically associated with active follicular growth and early CL development and function. Furthermore, the observation in yeast that deletion of Der1p, homolog of mammalian DERL1, abolished degradation of the substrate proteins [35] also adds more evidence that DERL1 plays a fundamental role in the cell and might be a pivotal factor in cell survival, which is consistent with its expression in growing follicles and functional CL. Finally, although the retrotranslocation pathways appear to require the function of the p97 ATPase complex [8], the present study found no interaction betwen p97 and DERL1 or between VIMP and DERL1. This could suggest that the binding of DERL1 to MHC I would be a regulation of MHC I molecules by DERL1 rather than a retrotranslocation process.

## Competing interests

The authors declare that they have no competing interests.

## Authors' contributions

KN participated in the design of the study, carried out the experiments described in the Methods section in the exception of the semi-quantitative RT-PCR, and drafted the manuscript. JLP participated in the design of the study and helped to draft the manuscript. JGL participated in the design of the study, performed the semi-quantitative RT-PCR experiments and helped drafting the manuscript. All authors read and approved the final manuscript.

## References

[B1] Ellgaard L Molinari M Helenius A Setting the standards: quality control in the secretory pathway Science 1999 286 5446 1882 1888 10.1126/science.286.5446.1882 10583943

[B2] Brodsky JL McCracken AA ER protein quality control and proteasome-mediated protein degradation Semin Cell Dev Biol 1999 10 5 507 513 10.1006/scdb.1999.0321 10597633

[B3] McCracken AA Brodsky JL Evolving questions and paradigm shifts in endoplasmic-reticulum-associated degradation (ERAD) Bioessays 2003 25 9 868 877 10.1002/bies.10320 12938176

[B4] Ploegh HL Viral strategies of immune evasion Science 1997 280 5361 248 253 10.1126/science.280.5361.248 9535648

[B5] Tortorella D Gewurz BE Furman MH Schust DJ Ploegh HL Viral subversion of the immune system Annu Rev Immunol 2000 18 861 926 10.1146/annurev.immunol.18.1.861 10837078

[B6] Wiertz EJ Jones TR Sun L Bogyo M Geuze HJ Ploegh HL The human cytomegalovirus US11 gene product dislocates MHC class I heavy chains from the endoplasmic reticulum to the cytosol Cell 1996 84 5 769 779 10.1016/S0092-8674(00)81054-5 8625414

[B7] Lilley BN Ploegh HL A membrane protein required for dislocation of misfolded proteins from the ER Nature 2004 429 6994 834 840 10.1038/nature02592 15215855

[B8] Ye Y Shibata Y Yun C Ron D Rapoport TA A membrane protein complex mediates retro-translocation from the ER lumen into the cytosol Nature 2004 429 6994 841 847 10.1038/nature02656 15215856

[B9] Fairchild DL Pate JL Interferon-gamma induction of major histocompatibility complex antigens on cultured bovine luteal cells Biol Reprod 1989 40 3 453 457 10.1095/biolreprod40.3.453 2503068

[B10] Benyo DF Haibel GK Laufman HB Pate JL Expression of major histocompatibility complex antigens on the bovine corpus luteum during the estrous cycle, luteolysis, and early pregnancy Biol Reprod 1991 45 2 229 234 10.1095/biolreprod45.2.229 1786287

[B11] Bukovsky A Caudle MR Keenan JA Wimalasena J Upadhyaya NB Van Meter SE Is corpus luteum regression an immune-mediated event? Localization of immune system components and luteinizing hormone receptor in human corpora lutea Biol Reprod 1995 53 6 1373 1384 10.1095/biolreprod53.6.1373 8562694

[B12] Petroff M Coggeshall KM Jones LS Pate JL Bovine luteal cells elicit major histocompatibility complex class II-dependent T-cell proliferation Biol Reprod 1997 57 4 887 893 10.1095/biolreprod57.4.887 9314594

[B13] Cannon MJ Davis JS Pate JL Expression of costimulatory molecules in the bovine corpus luteum Reprod Biol Endocrinol 2007 5 5 17266770 10.1186/1477-7827-5-5 PMC1800853

[B14] Cannon MJ Petroff MG Pate JL Effects of prostaglandin F2alpha and progesterone on the ability of bovine luteal cells to stimulate T lymphocyte proliferation Biol Reprod 2003 69 2 695 700 10.1095/biolreprod.103.017590 12724272

[B15] Ndiaye K Poole DH Pate JL Expression and regulation of functional oxytocin receptors in bovine T lymphocytes Biol Reprod 2008 78 4 786 793 10.1095/biolreprod.107.065938 18094352

[B16] Cannon MJ Pate JL The role of major histocompatibility complex molecules in luteal function Reprod Biol Endocrinol 2003 1 93 10.1186/1477-7827-1-93 14613531 PMC293428

[B17] Ndiaye K Fayad T Silversides DW Sirois J Lussier JG Identification of downregulated messenger RNAs in bovine granulosa cells of dominant follicles following stimulation with human chorionic gonadotropin Biol Reprod 2005 73 2 324 333 10.1095/biolreprod.104.038026 15829623

[B18] Brûlé S Rabahi F Faure R Beckers JF Silversides DW Lussier JG Vacuolar system-associated protein-60: a protein characterized from bovine granulosa and luteal cells that is associated with intracellular vesicles and related to human 80K-H and murine beta-glucosidase II Biol Reprod 2002 62 3 642 654 10.1095/biolreprod62.3.642 10684806

[B19] Bédard J Brûlé S Price CA Silversides DW Lussier JG Serine protease inhibitor-E2 (SERPINE2) is differentially expressed in granulosa cells of dominant follicle in cattle Mol Reprod Dev 2003 64 2 152 165 10.1002/mrd.10239 12506347

[B20] Rabahi F Brûlé S Sirois J Beckers JF Silversides DW Lussier JG High expression of bovine alpha glutathione S-transferase (GSTA1, GSTA2) subunits is mainly associated with steroidogenically active cells and regulated by gonadotropins in bovine ovarian follicles Endocrinology 1999 140 8 3507 3517 10.1210/en.140.8.3507 10433206

[B21] Bradford MM A rapid and sensitive method for the quantitation of microgram quantities of protein utilizing the principle of protein-dye binding Anal Biochem 1976 72 248 254 10.1016/0003-2697(76)90527-3 942051

[B22] Brûlé S Faure R Doré M Silversides DW Lussier JG Immunolocalization of vacuolar system-associated protein-60 (VASAP-60) Histochem Cell Biol 2003 119 5 371 381 12750905 10.1007/s00418-003-0521-8

[B23] Sirois J Richards JS Transcriptional regulation of the rat prostaglandin endoperoxide synthase 2 gene in granulosa cells. Evidence for the role of a cis-acting C/EBP beta promoter element J Biol Chem 1993 268 29 21931 21938 8408049

[B24] Espey LL Ujioka T Russell DL Skelsey M Vladu B Robker RL Okamura H Richards JS Induction of early growth response protein-1 gene expression in the rat ovary in response to an ovulatory dose of human chorionic gonadotropin Endocrinology 2000 141 7 2385 2391 10.1210/en.141.7.2385 10875238

[B25] Eppig JJ Wigglesworth K Pendola FL The mammalian oocyte orchestrates the rate of ovarian follicular development Proc Natl Acad Sci USA 2002 99 5 2890 2894 10.1073/pnas.052658699 11867735 PMC122443

[B26] Ochsner SA Day AJ Rugg MS Breyer RM Gomer RH Richards JS Disrupted function of tumor necrosis factor-alpha-stimulated gene 6 blocks cumulus cell-oocyte complex expansion Endocrinology 2003 144 10 4376 4384 10.1210/en.2003-0487 12959984

[B27] Wang J Hua H Ran Y Zhang H Liu W Yang Z Jiang Y Derlin-1 is overexpressed in human breast carcinoma and protects cancer cells from endoplasmic reticulum stress-induced apoptosis Breast Cancer Res 2008 10 1 R7 10.1186/bcr1849 18205950 PMC2374959

[B28] Ye Y Meyer HH Rapoport TA Function of the p97-Ufd1-Npl4 complex in retrotranslocation from the ER to the cytosol: dual recognition of nonubiquitinated polypeptide segments and polyubiquitin chains J Cell Biol 2003 162 1 71 84 10.1083/jcb.200302169 12847084 PMC2172719

[B29] Ye Y Shibata Y Kikkert M van Voorden S Wiertz E Rapoport TA Inaugural Article: Recruitment of the p97 ATPase and ubiquitin ligases to the site of retrotranslocation at the endoplasmic reticulum membrane Proc Natl Acad Sci USA 2005 102 40 14132 14138 10.1073/pnas.0505006102 16186510 PMC1242302

[B30] Penny LA Armstrong D Bramley TA Webb R Collins RA Watson ED Immune cells and cytokine production in the bovine corpus luteum throughout the oestrous cycle and after induced luteolysis J Reprod Fertil 1999 115 1 87 96 10.1530/jrf.0.1150087 10341726

[B31] Baker BM Tortorella D Dislocation of an endoplasmic reticulum membrane glycoprotein involves the formation of partially dislocated ubiquitinated polypeptides J Biol Chem 2007 282 37 26845 26856 10.1074/jbc.M704315200 17650499

[B32] Loureiro J Lilley BN Spooner E Noriega V Tortorella D Ploegh HL Signal peptide peptidase is required for dislocation from the endoplasmic reticulum Nature 2006 441 7095 894 897 10.1038/nature04830 16738546

[B33] Lilley BN Ploegh HL Multiprotein complexes that link dislocation, ubiquitination, and extraction of misfolded proteins from the endoplasmic reticulum membrane Proc Natl Acad Sci USA 2005 102 40 14296 14301 10.1073/pnas.0505014102 16186509 PMC1242303

[B34] Altman A Coggeshall KM Mustelin T Molecular events mediating T cell activation Adv Immunol 1990 48 227 360 full_text 1693465 10.1016/s0065-2776(08)60756-7

[B35] Knop M Finger A Braun T Hellmuth K Wolf DH Der1, a novel protein specifically required for endoplasmic reticulum degradation in yeast EMBO J 1996 15 4 753 763 8631297 PMC450274

